# Yellow nutsedge *WRI4*-like gene improves drought tolerance in *Arabidopsis thaliana* by promoting cuticular wax biosynthesis

**DOI:** 10.1186/s12870-020-02707-7

**Published:** 2020-10-31

**Authors:** Chao Cheng, Shutong Hu, Yun Han, Di Xia, Bang-Lian Huang, Wenhua Wu, Jamshaid Hussain, Xuekun Zhang, Bangquan Huang

**Affiliations:** 1 State Key Laboratory of Biocatalysis and Enzyme Engineering, College of Life Science, Hubei University, Wuhan, 430062 China; 2 Biotechnology Department, COMSATS University Islamabad, Abbottabad Campus 22060, University Road, Abbottabad, Pakistan; 3 Hubei Key Laboratory of Waterlogging Disaster and Agricultural Use of Wetland, Yangtze University, Jingzhou, 434023 China

**Keywords:** *Cyperus esculentus*, *WRI4*-like gene, Cuticular wax biosynthesis, Drought tolerance, Gene expression

## Abstract

**Background:**

Cuticular wax plays important role in protecting plants from drought stress. In Arabidopsis *WRI4* improves drought tolerance by regulating the biosynthesis of fatty acids and cuticular wax. *Cyperus esculentus* (yellow nutsedge) is a tough weed found in tropical and temperate zones as well as in cooler regions. In the current study, we report the molecular cloning of a *WRI4*-like gene from *Cyperus esculentus* and its functional characterization in Arabidopsis.

**Results:**

Using RACE PCR, full-length *WRI*-like gene was amplified from yellow nutsedge. Phylogenetic analyses and amino acid comparison suggested it to be a *WRI4*-like gene. According to the tissue-specific expression data, the highest expression of *WRI4*-like gene was found in leaves, followed by roots and tuber. Transgenic Arabidopsis plants expressing nutsedge *WRI4*-like gene manifested improved drought stress tolerance. Transgenic lines showed significantly reduced stomatal conductance, transpiration rate, chlorophyll leaching, water loss and improved water use efficiency (WUE). In the absence of drought stress, expression of key genes for fatty acid biosynthesis was not significantly different between transgenic lines and WT while that of cuticular wax biosynthesis genes was significantly higher in transgenic lines than WT. The PEG-simulated drought stress significantly increased expression of key genes for fatty acid as well as wax biosynthesis in transgenic Arabidopsis lines but not in WT plants. Consistent with the gene expression data, cuticular wax load and deposition was significantly higher in stem and leaves of transgenic lines compared with WT under control as well as drought stress conditions.

**Conclusions:**

*WRI4*-like gene from *Cyperus esculentus* improves drought tolerance in Arabidopsis probably by promoting cuticular wax biosynthesis and deposition. This in turn lowers chlorophyll leaching, stomatal conductance, transpiration rate, water loss and improves water use efficiency under drought stress conditions. Therefore, *CeWRI4*-like gene could be a good candidate for improving drought tolerance in crops.

## Background

Aerial surfaces of terrestrial plants are covered with a layer of cuticular wax, which protects plants from pathogen infection [1], insect attack [2], UV radiation [3, 4] and drought stress [5–10]. Cuticular wax is a complex mixture consisting mainly of very-long-chain fatty acids (VLCFAs) and their derivatives [11–13]. First C16 and C18 fatty acids are synthesized in plastids and then hydrolyzed and exported to the cytoplasm. The synthesized C16 and C18 coenzyme As (CoAs) are elongated into VLCFAs and then modified into primary alcohols and wax esters by alcohol-forming pathway, or into aldehydes, alkanes, secondary alcohols and ketones by alkane-forming pathway [14, 15]. The regulation of cuticular wax biosynthesis takes place at transcriptional [6, 14, 16–19], posttranscriptional [20, 21] and posttranslational levels [22]. Many attempts have been made to increase the cuticular wax content in plant organs with an ultimate objective of improving drought stress tolerance [6, 16, 18, 23–26].

The AP2 (APETALA2)/EREBP (Ethylene Responsive Element Binding Protein) transcription factors contain either a single or two AP2/ERF domains and are involved in various biotic and abiotic stresses response [27]. Arabidopsis WRI1 gene, cloned by Cernac and Benning (2004), was placed in AP2 subfamily since it contained two AP2/EREB domains [28]. The WRI1-like group consists of four members, namely WRI1 (At3g54320), WRI2 (At2g41710), WRI3 (At1g16060) and WRI4 (At1g79700). Previously the WRI1-like group was considered to be part of the ANT-like group [29], while others regarded it as a monophyletic group of the AP2 subfamily [30]. Three members of WRI1-like group, namely WRI1, WRI3 and WRI4, play role in activation of fatty acid biosynthesis [28, 30], while the fourth member WRI4 is involved in drought tolerance in Arabidopsis through regulation of cuticular wax biosynthesis in stem [10].

*Cyperus esculentus* (yellow nutsedge), a crop of the sedge family, is widespread across much of the globe. It is considered a crop native to areas in Africa and tropical Asia [31–33]. It is also found in tropical and subtropical regions of Asia, Europe, Africa and North America [34, 35]. The tubers and seeds of yellow nutsedge can easily be dispersed and get mixed up with crop seeds [36–38]. As its tubers are enriched in sugar, protein, starch, fibers, minerals, vitamins and oil, efforts are underway to develop yellow nutsedge as a new oil crop [39–41]. In the current study we report the cloning and characterization of a *WRI4*-like gene from *Cyperus esculentus (CeWRI4)*. The gene was introduced in Arabidopsis for its functional characterization.

## Results and discussions

### WRI4 nature of the nutsedge gene

In this study, first a 490 bp fragment corresponding to the conserved region of nutsedge *WRI*-like gene was amplified through RT-PCR by using degenerated primers (Additional file 1). Subsequently, a 1423 bp full length fragment was obtained by using the 5′- and 3′-RACE PCRs (Additional file 1). The amplified gene contained an ORF of 1098 bp, 5’UTR (untranslated region) and 3’UTR (Additional file 2). Conserved domain analysis indicated that this WRI-like gene contained two AP2 domains, with the second domain being more conserved than the first one as shown by multiple alignment analysis (data not shown). This sequence is deposited in NCBI Genbank as accession MW039149.

AP2/EREB genes are classified into five groups, namely AP2 subfamily with two AP2/ERF domains, DREB and ERF subfamilies with one AP2/ERF domain, RAV subfamily with one AP2/ERF domain and B3 DNA-binding motif, and others [42–44]. In this study the two AP2 domain-containing genes clustered into three major groups: AP2, ANT and WRI. In the WRI group the WRI1s were clearly separated from WRI2s, WRI3s and WRI4s, and the nutsedge WRI-like gene was found to be more closely related to WRI3s and WRI4s (Additional file 3). Amino acid sequence comparison indicated that this nutsedge WRI-like peptide showed 31.06 and 52.03% amino acid sequence similarity with Arabidopsis WRI3 and WRI4, respectively, therefore it was named as *C. esculentus WRI4* (*CeWRI4*). We then determined the expression of *CeWRI4* in different organs. *CeWRI4* showed the highest expression in leaves, followed by roots and tubers (Additional file 4).

### The expression of *CeWRI4* improved drought tolerance in transgenic Arabidopsis

**Fig. 1:**
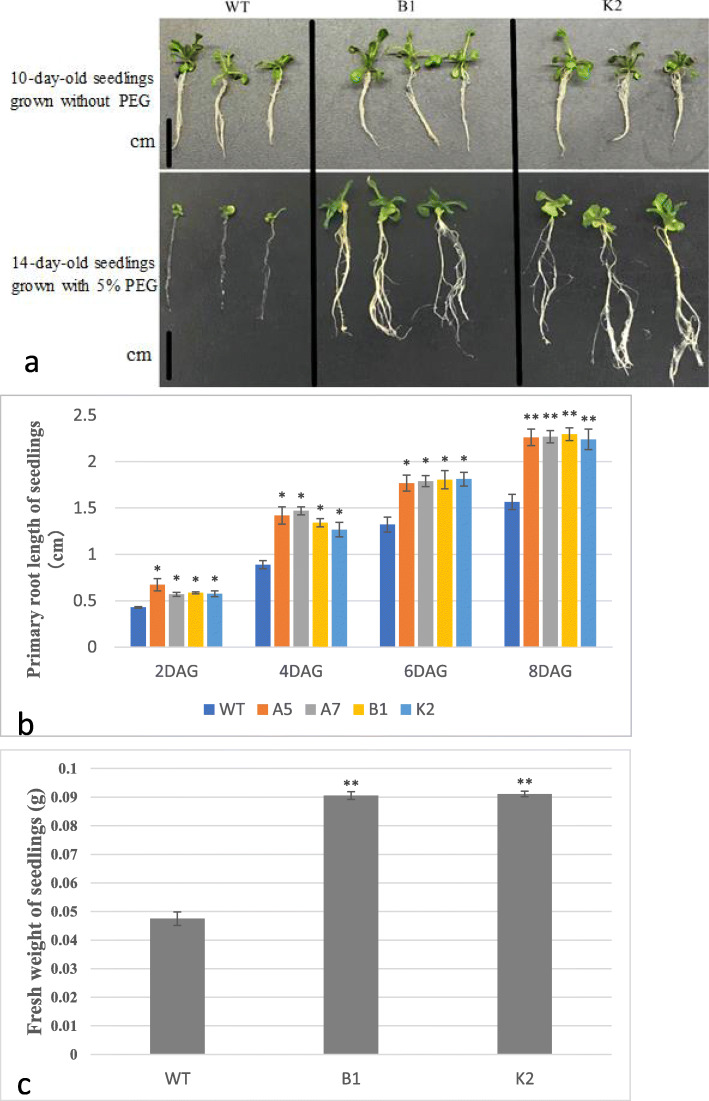
**a**: 10-day-old WT and transgenic lines B1 and K2 seedlings without PEG stress (up) and 14-day-old seedlings of WT and transgenic lines B1 and K2 with PEG stress (down). Scale bar = 1 cm; **b**: Primary root length of seedlings from WT and transgenic lines A5, A7, B1 and K2 grown on 1/2 MS agar medium added with 5% PEG-6000; **c**: Fresh weight of 14-day -old seedlings from WT and transgenic lines A5, A7, B1 and K2 grown on 1/2 MS agar medium added with 5% PEG-6000; Error bars indicate ±SD, **P < 0.01, *P < 0.05

For functional characterization of the cloned gene, *Arabidopsis thaliana* plants were transformed with *CeWRI4* and drought tolerance of transgenic plants was determined by growing on PEG-supplemented growth media as well as by applying real dehydration. Seed germination of both WT and transgenic Arabidopsis was higher than 90% in both control and PEG-simulated stress conditions (data not shown). We also determined drought tolerance at seedling stage. In control conditions, the growth of 10-day-old WT seedlings was not significantly different from transgenic lines B1 and K2 (Fig. 1a). However, under PEG-induced drought stress, WT seedlings exhibited severe inhibition of primary root growth and much fewer lateral roots compared with transgenic lines (Fig. 1a). Under drought stress condition transgenic lines had more number of leaves than WT. Two-week-old transgenic plants had 4 well-developed true leaves compared with two very small and yellow true leaves in same-aged WT plants (Fig. 1a). The root length and seedling fresh weight (FW) data also confirmed that the transgenic lines exhibited higher tolerance to PEG-simulated drought stress (Fig. 1b-c).

**Fig. 2:**
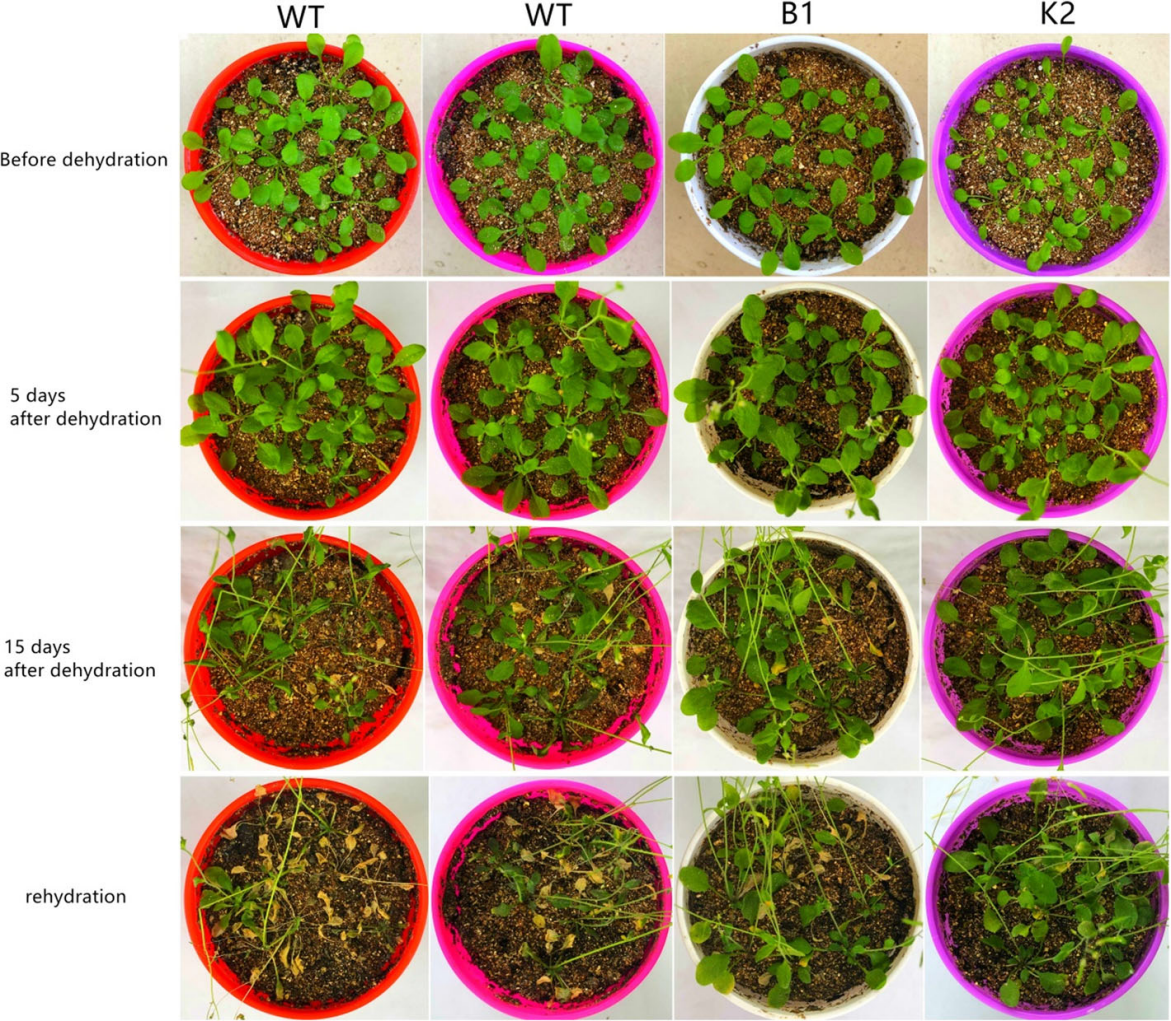
Morphology of WT and transgenic lines B1 and K2 6 days before dehydration; 5 days and 15 days after dehydration; 1 day after rehydration. Before drought stress the plants were well watered

**Fig. 3:**
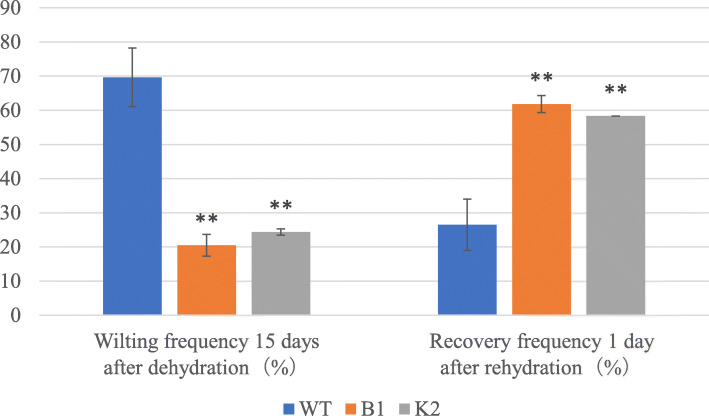
Wilting frequency after 15 days of dehydration and recovery frequency 1 day after rehydration. The recovery frequency was calculated as survived plants to all plants scored; Error bars indicate ±SD, **P < 0.01

We also determined the plant drought tolerance through real dehydration by withholding water. The growth of WT and transgenic Arabidopsis plants was not significantly different under normal growth conditions (Fig. 2). However, after 15 days of dehydration, the wilting frequency was 63.6–81.8% in WT compared with 18.2–25% in transgenic lines (Figs. 2 and 3). After 22 days of dehydration, the stressed plants were re-watered, and the extent of recovery was determined 1 day after resumption of watering. The transgenic lines showed significantly higher recovery frequency, i.e., 58.3–63.6% compared with 18.1–36.3% in WT (Figs. 2 and 3). Based on these data, it can be concluded that *CeWRI4* improves drought stress tolerance in Arabidopsis.

### Gas exchange parameters and water relations were altered in transgenic Arabidopsis lines

**Fig. 4:**
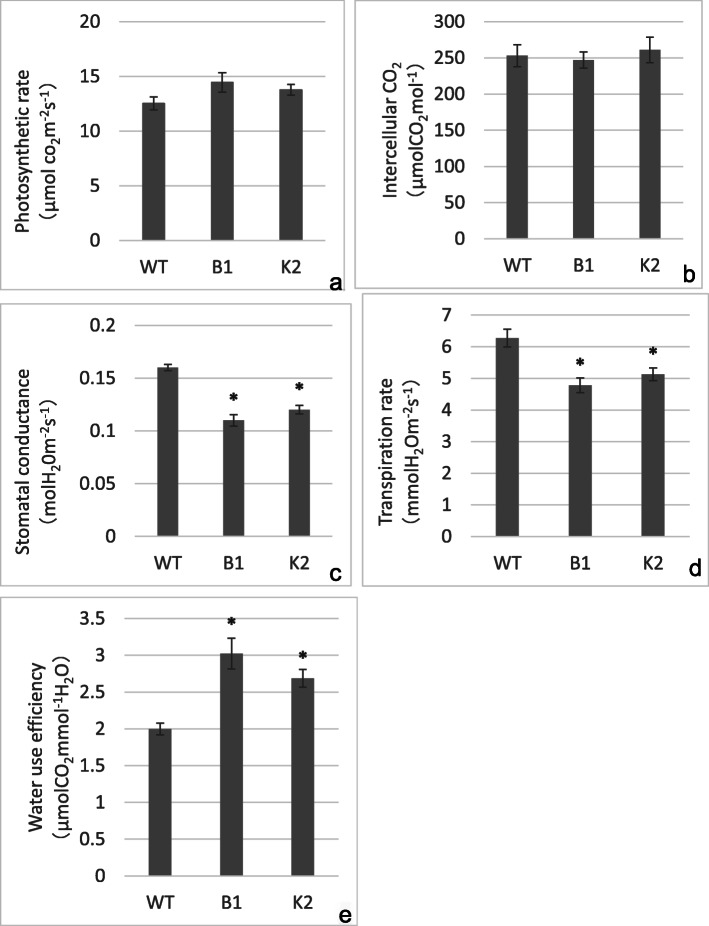
Photosynthesis-related parameters measurement. 5-week-old transgenic and wild type plants grown under normal conditions were treated with 5% PEG-6000 for 1 day. Parameters were recorded by photosynthetic system instrument at the same position of each plant. **a**: Photosynthetic rate of single leaf from WT, B1 and K2 plants; **b**: Intercellular CO_2_ content of single leaf from WT, B1 and K2 plants; **c**: Stomatal conductance of single leaf from WT, B1 and K2 plants; **d**: Transpiration rate of single leaf from WT, B1 and K2 plants; **e**: Water use efficiency of single leaf from WT, B1 and K2 plants. Error bars indicate ±SD, *P < 0.05

In plants more than 90% of water uptake is lost through transpiration, mainly via stomata [45]. It is well documented that stomata closure is one of the first responses of plants to drought in order to avoid excessive water loss and to protect the photosynthetic machinery [46]. Control of stomatal conductance under drought is considered to be a promising approach for developing drought resistance in crops [47]. Transgenic soybean overexpressing Arabidopsis *LOS5/ABA3* exhibited reduced water loss by decreasing stomatal aperture and transpiration rate, thereby alleviating leaf wilting and maintained higher relative water content [48]. Transgenic rice expressing *AtEDT1/HDG11* also exhibited increased WUE and photosynthesis by reducing stomatal conductance and transpiration [49]. In this study it was found that net photosynthesis and intercellular CO_2_ concentration were not significantly different between WT and transgenic lines when drought stressed (Fig. 4a-b). On the other hand, transgenic lines showed significantly lower stomatal conductance and transpiration rate compared with WT (Fig. 4c-e). Under drought stress, the stomatal conductance of transgenic B1 and K2 was 69 and 75% of WT, respectively (Fig. 4c), while the transpiration rate was 76 and 82% of WT, respectively (Fig. 4d). Transgenic lines exhibited higher water use efficiency, which was 150 and 130% of WT in transgenic lines B1 and K2, respectively (Fig. 4e). The data reported in our study is in good agreement with previous reports [50–52].

### Transgenic Arabidopsis plants showed slower chlorophyll leaching and water loss

**Fig. 5:**
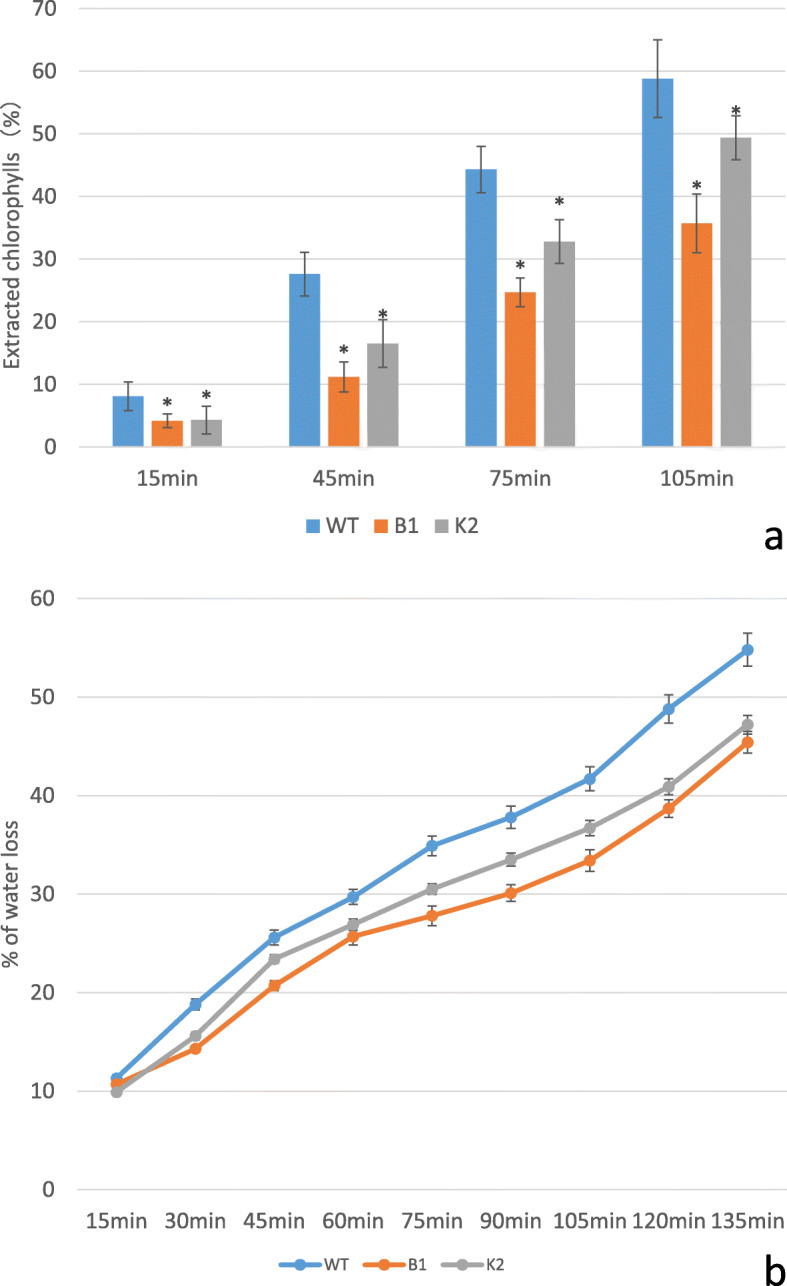
Chl extraction and water loss experiment. **a**: Chl extraction. Data were shown as percentage of total chlorophyll content 24 h after initial soaking; **b**: Water loss assays. Percentage of water loss was calculated as that follows: (W1-W2)/W1 (W1: Initial leaf weight before water loss; W2: Leaf weight after water loss at different time points). Error bars indicate ±SD, *P < 0.05

Leaves of WT and transgenic Arabidopsis seedlings were immersed in 80% ethanol to determine the chlorophyll leaching. As the time went on, more and more chlorophyll leached out in both WT and transgenic lines, however the latter showed significantly slower chlorophyll leaching compared with WT. After 105 mins, chlorophyll leaching for B1 and K2 lines was lower than 50 and 40%, respectively, while in WT it was higher than 60% (Fig. 5a). The water loss from detached leaves was lower than 50% in transgenic lines compared with 58% in WT after 135 mins (Fig. 5b).

### Expression of key genes for fatty acid and cuticular wax biosynthesis was altered in transgenic Arabidopsis lines

Cuticular wax plays important roles in protecting plants from drought stress [6–10, 53, 54]. It was shown that WRI4 activates the expression of genes involved in fatty acid elongation of wax precursors and production of wax esters by directly binding to their promoters. WRI4 also plays role in fatty acid biosynthesis by directly binding to promoters of genes like *BCCP1* and *BCCP2* [10, 30]. Disruption of *WRI4* led to down-regulation of above-mentioned genes and other fatty acid biosynthesis genes such as *PKP1*, *PKP2*, *PDHE1α* and *ENR1* [10].

**Fig. 6:**
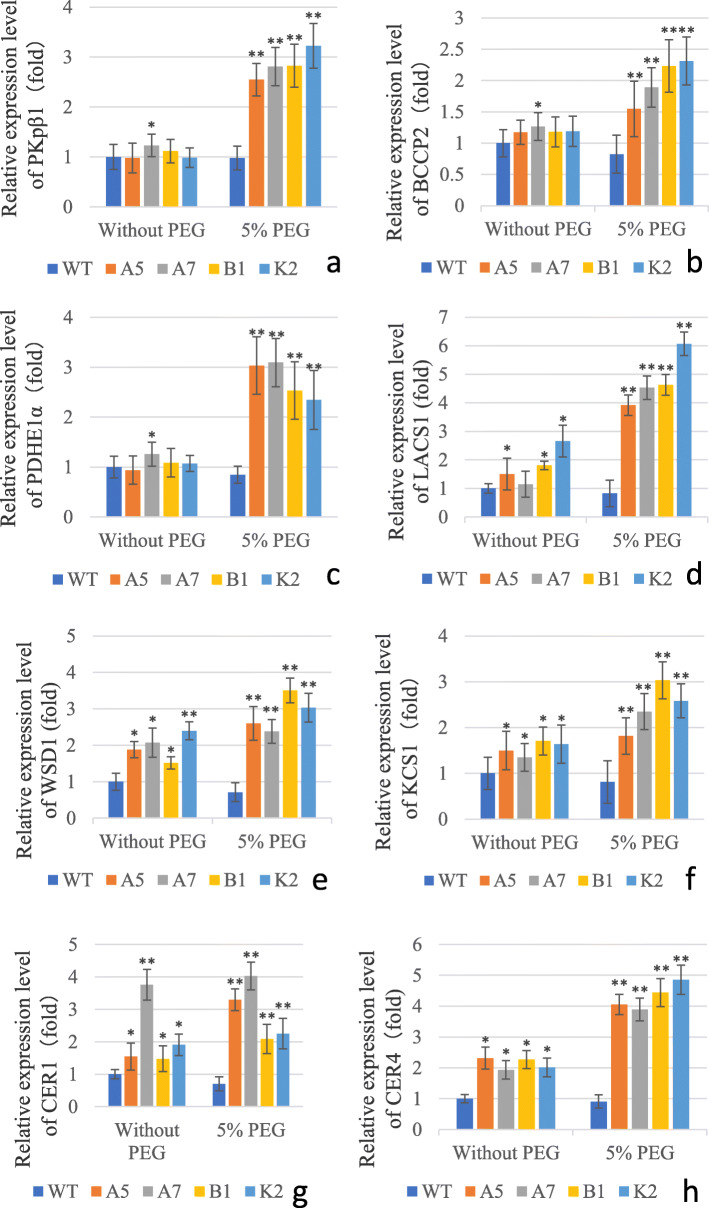
qRT-PCR analysis of genes involved in fatty acids and cuticular wax biosynthesis in WT and transgenic lines A5, A7, B1, K2. Each value was calculated as the average of three independent measurements. Error bars indicate ±SD, **P < 0.01, *P < 0.05

Keeping in view the role of *CeWRI4*, and to further understand the mechanism of drought tolerance in transgenic lines, we attempted to explore the expression of key genes involved in fatty acid and cuticular wax biosynthesis. Quantitative RT-PCR data indicated that under control growth conditions the expression of fatty acid biosynthesis genes such as *PKp-β1* (plastidic pyruvate kinase beta subunit 1, At5g52920), *BCCP2* (biotin carboxyl carrier protein 2, At5g15530) and *PDHE1α* (pyruvate dehydrogenase E1 alpha, At1g01090) [28], was not significantly different between WT and transgenic Arabidopsis lines. In WT plants, PEG-induced drought stress did not significantly alter the expression of above-mentioned genes as compared with its control. On the other hand, expression of *PKp-β1*, *BCCP2* and *PDHE1α* was significantly induced by PEG treatment (130–230%, 50–100% and 130–220% increase, respectively) in transgenic lines as compared with the respective unstressed transgenic lines (Fig. 6a-c).

We also determined the expression of a few key cuticular wax biosynthesis genes, namely *LACS1* (long-chain acyl-coa synthase 1, At2g47240), *WSD1* (wax ester synthase/diacylglycerol acyltransferase 1, At5g37300), *KCS1* (3-ketoacyl-CoA synthase 1, At1g01120), *CER1* (ECERIFERUM 1, At1g02200) and *CER4* (ECERIFERUM 4, At4g33790) [30–33]. Under normal growth conditions, expressions of all the above mentioned genes was significantly higher in the transgenic lines compared with WT. PEG treatment did not cause significant change in expression of these gene in WT, however, in transgenic lines the gene expression was significantly induced by PEG (gene expression being 130–300%, 30–80%, 20–80%, 10–110%, 80–140% higher for *LACS1*, *WSD1*, *KCS1*, *CER1* and *CER4,* respectively, Fig. 6d-h). We also determined the expression of *AtWRI4*, a transcription factor regulating wax biosynthesis, in transgenic Arabidopsis. Under control as well as drought stress conditions, the expression of *AtWRI4* was not significantly different between WT and transgenic lines (Additional file 5). The expression of *DGAT1* (At2g19450), a gene involved in TAG accumulation [29], was significantly lower (10–40% decrease) in transgenic lines compared with WT in unstressed conditions. PEG-induced drought stress did not cause significant change in expression of *DGAT1* in WT, but a significant decrease (20–70%) in expression of this gene was observed in the transgenic lines compared with both WT and transgenic control (Additional file 6). Overall, these data showed that expression of fatty acid, cuticular wax and oil synthesis genes is significantly modulated in transgenic Arabidopsis lines expressing *CeWRI4*.

### Cuticular wax load but not oil content was higher in transgenic Arabidopsis lines

Cuticular wax is important for preventing non-stomatal water loss from the aerial parts of terrestrial plants. It is, therefore, closely correlated with plant drought resistance [9, 55]. Overexpression of genes like *SHN1/WIN1*, *SHINE1*, *MdSHINE2, WXP1, WXP2, WSD1, MYB96* and *MYB94* increased drought tolerance by increasing cuticular wax biosynthesis and deposition [6–8, 16, 18, 23–26, 56]. Alkane biosynthesis is one of the key responses of plants under osmotic stress conditions [9]. It has been proposed that cuticular alkanes confer greater resistance to water diffusion than VLCFAs [9, 16]. Seo et al. [16] overexpressed *MYB96* in Arabidopsis which resulted in ~ 8.6-fold and ~ 1.6-fold increase in total wax load in transgenic leaves and stems, respectively, compared with WT. It was further shown that increase in wax was mainly due to elevated alkanes. Similarly, transgenic mulberry overexpressing *AtSHINE1* also showed increased leaf surface wax load mainly due to increased alkanes in the wax components [25].

**Fig. 7:**
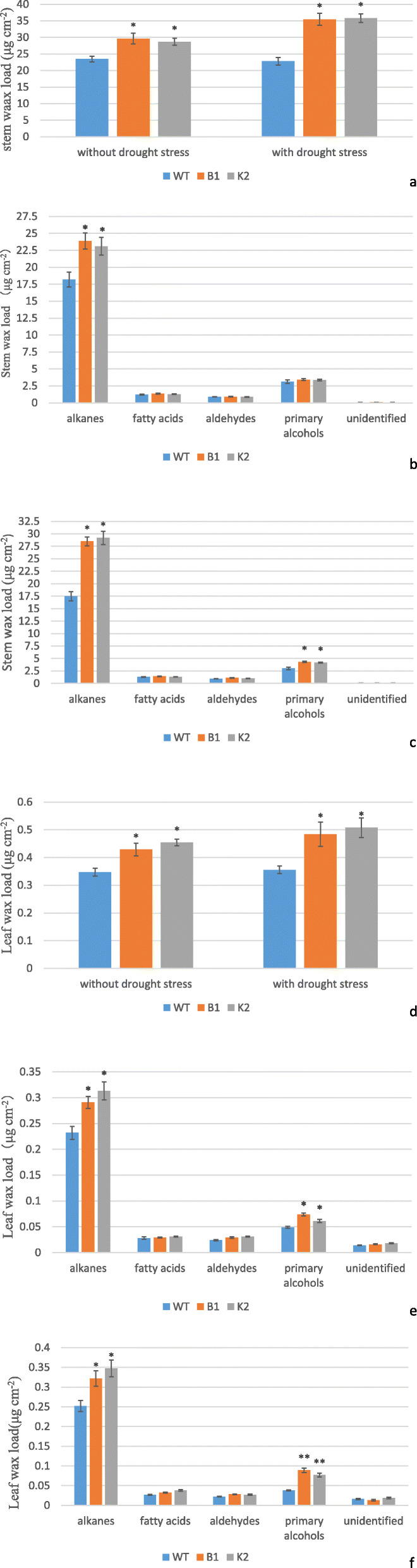
Cuticular wax content and composition stems and leaves of WT and transgenic lines B1 and K2. **a**: Total wax load in stems of WT and transgenic lines B1 and K2 under drought stress and non-stress conditions; **b**: Cuticular wax compositions in stems of WT and transgenic lines B1 and K2 under non-stress condition; **c**: Cuticular wax compositions in stems of WT and transgenic lines B1 and K2 under drought-stress condition; **d**: Total wax load in leaves of WT and transgenic lines B1 and K2 under drought stress and non-stress conditions; **e**: Cuticular wax compositions in leaves of WT and transgenic lines B1 and K2 under non-stress condition. **f**: Cuticular wax compositions in leaves of WT and transgenic lines B1 and K2 under drought-stress condition. Error bars indicate ±SD, *P < 0.05

In this study it was found that without drought stress, stem wax content was higher by 26 and 22% in transgenic lines B1 and K2, respectively, than WT (Fig. 7a). Among the wax components, alkanes were higher in transgenic lines than WT (31 and 26% increase in transgenic lines B1 and K2, respectively), while other cuticular wax components were not significantly different between transgenic lines and WT (Fig. 7b). Similarly, total leaf wax load was higher by 24 and 31%, respectively, in transgenic lines B1 and K2 than WT (Fig. 7d). Of the leaf waxes, alkanes were 25 and 35% higher while primary alcohols were 51 and 1.24% higher in transgenic lines B1 and K2, respectively, than WT. Other cuticular wax components such as aldehydes and fatty acids were not significantly different (Fig. 7e).

With drought stress stem wax load was significantly increased in transgenic B1 and K2, while leaf wax load was significantly increased in K2 but not in B1 (Fig. 7a, d). It was found that with drought stress total wax in stems of transgenic B1 and K2 was 55 and 57% higher than that in WT (Fig. 7a), in which the alkanes were 63 and 67% higher in transgenic B1 and K2 than in WT, respectively, while primary alcohols were 43 and 38% higher than that in WT leaves, respectively, and other cuticular wax components were not significantly different (Fig. 7c). Similarly, total wax load was 36 and 43% higher in transgenic B1 and K2 leaves than in WT (Fig. 7d), in which the alkanes were 28 and 38% higher in transgenic B1 and K2 than in WT, respectively, while primary alcohols were 134 and 102% higher than that in WT leaves, respectively, and other cuticular wax components such as aldehydes and fatty acids were not significantly different (Fig. 7f).

Oil content in Arabidopsis leaves was also assayed, however no significant difference was found between WT and transgenic lines (Additional file 7). This is consistent with the *DGAT1* expression data. The expression of *DGAT1* was significantly lower in transgenic lines compared with WT under drought stress conditions (Additional file 6). This could be the possible reason for no significant difference in oil content between WT and transgenic lines, even though the expression of genes involved in fatty acid biosynthesis was significantly higher in transgenic lines than WT (Fig. 6a-c).

### Epicuticular wax crystal deposition was higher in transgenic Arabidopsis leaves

**Fig. 8:**
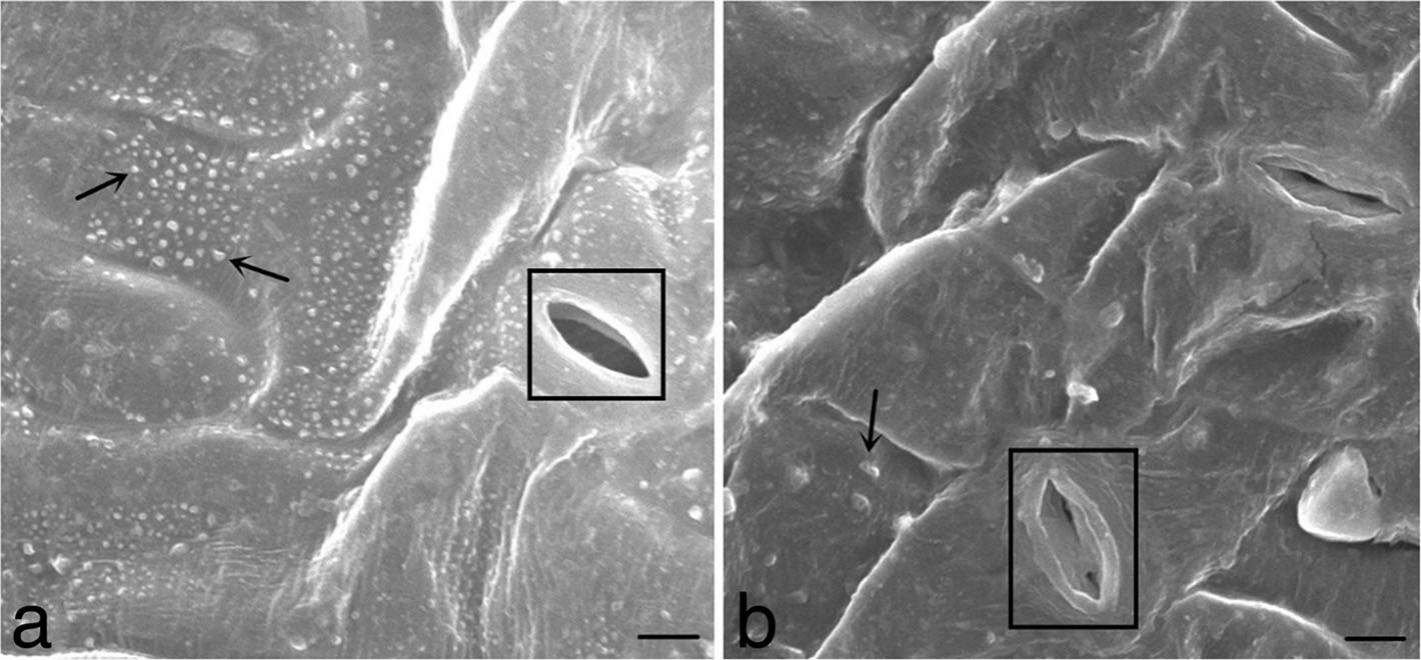
SEM images of wax crystals in adaxial epidermis of 5-week-old transgenic (**a**) and WT (**b**) Arabidopsis plants after 10 days’ drought stress. Epicuticular wax crystals indicated by arrows and stoma by rectangular boxes. Scale bar = 5 μm

Scanning electron microscopic (SEM) analysis indicated that significantly more epicuticular wax crystals were observed on leaf surface of transgenic Arabidopsis under drought stress condition compared with WT (Fig. 8a-b). The stoma in transgenic lines remained smooth and normal while shrinking and distortion was observed in WT stoma (Fig. 8a-b). These data indicate that *CeWRI4* expression in Arabidopsis significantly increased the leaf epicuticular wax crystal deposition.

### Transgenic Arabidopsis plants showed significantly lower soluble sugars, free proline and MDA content under drought stress

**Fig. 9:**
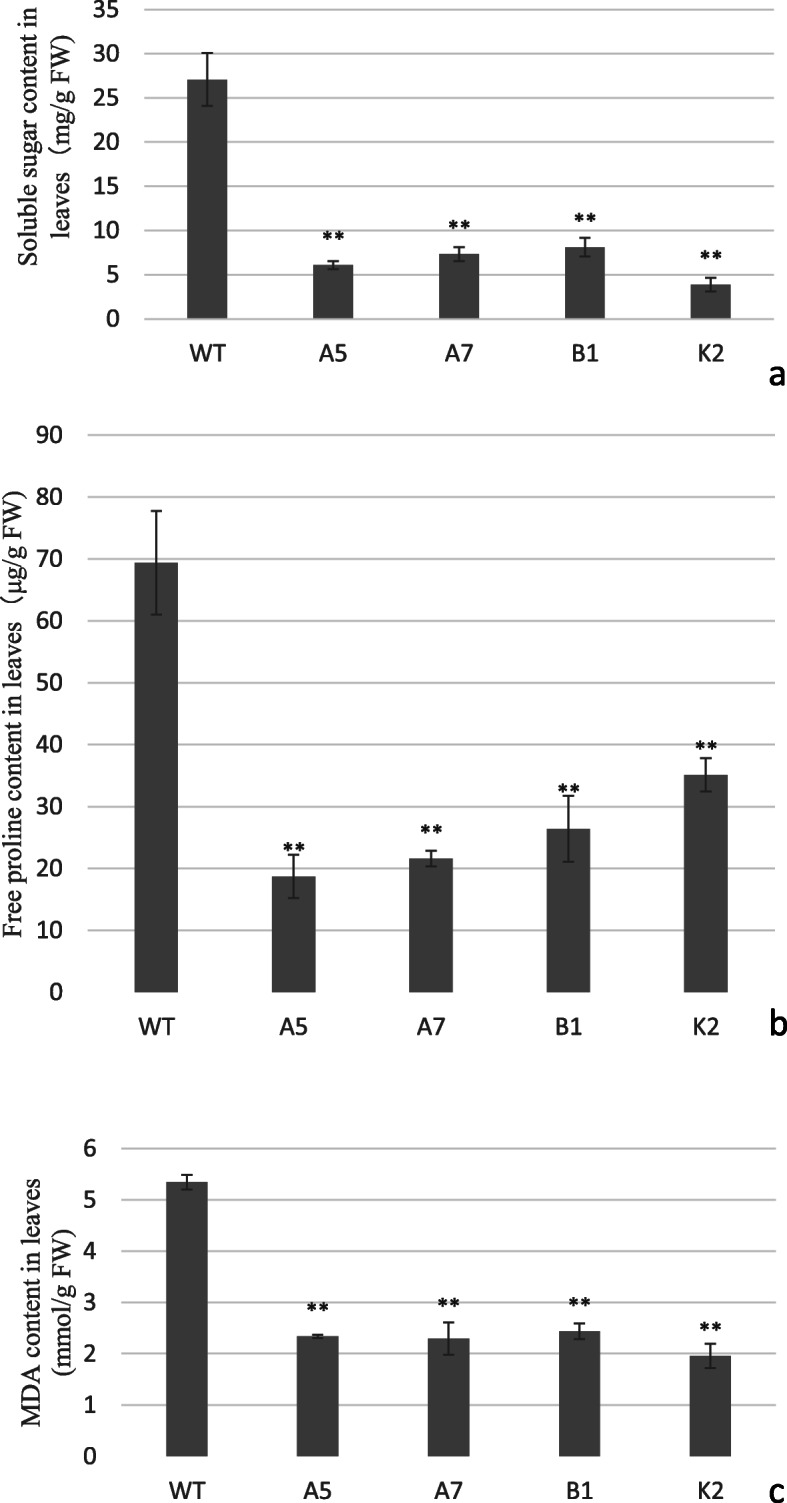
Physiological indices in WT and transgenic Arabidopsis lines A5, A7, B1 and K2 grown with water deprivation. **a** Soluble sugar content in leaves; **b** Free proline content in leaves; C. MDA content in leaves. Error bars indicate ±SD, **P < 0.01

Malondialdehyde (MDA), the product of membrane peroxidation, is considered an indicator of lipid peroxidation and membrane damage [51, 57, 58]. It has been observed that plants, when subjected to drought stress, accumulate osmolytes such as soluble sugars and proteins to maintain osmotic equilibrium and membrane integrity [59, 60]. Many plants modulate their osmotic adjustment abilities to resist drought stress via accumulating proline and soluble sugars, which participate in osmotic protection [61, 62]. To gain further insights into drought tolerance phenotype of transgenic Arabidopsis expressing *CeWRI4,* we assayed the content of soluble sugars, free proline and MDA. After two weeks of real dehydration, the concentration of soluble sugars was significantly lower in transgenic lines as compared with WT (only 14.3–29.9% of WT, Fig. 9a). A similar trend was observed for proline and MDA content. In transgenic lines proline and MDA were also significantly lower as compared with WT (being 26.9–50.6% and 36.5–45.5% of WT contents, respectively, Fig. 9b-c). These results suggested that probably the transgenic lines suffered less lipid peroxidation and membrane damage.

## Conclusions

In this study we cloned a *WRI4*-like gene from *C. esculentus* and functionally characterized it by expressing it in Arabidopsis. *CeWRI4*-like gene improved drought tolerance in Arabidopsis by promoting cuticular wax biosynthesis and deposition, thus lowering chlorophyll leaching, stomatal conductance, transpiration rate, water loss and increasing water use efficiency under drought stress. *CeWRI4* could be a valuable target for genetic manipulation to improve drought tolerance of commercially important crops.

## Methods

### Plant materials and treatments

*Arabidopsis thaliana* ecotype Columbia and tubers of *Cyperus esculentus* (yellow nutsedge) cv. Hubu-1 were provided by State Key Laboratory of Biocatalysis, College of Life Sciences, Hubei University, China. The yellow nutsedge tubers were germinated at 22 °C under a 16 h light/8 h dark cycle. RNA was isolated from leaves, roots and fresh tubers as described by Cheng et al. [63].

### Cloning of *WRI4* from yellow nutsedge

WRI1 protein sequences from different plant species (*Solanum tuberosum*, accession No.: AAA66057; *Nicotiana tabacum*, accession No.: ABD60582; *Fragaria x ananassa*, accession No.: AAS00541; Gladiolus hybrid cultivar, accession No.: AHN15416; *Triticum aestivum*, accession No.: AAF61173; *Hordeum vulgare*, accession No.: AAU06191; https://www.ncbi.nlm.nih.gov/) were aligned by ClustalW2 (http://www.ebi.ac.uk/Tools/msa/clustalw2/). Degenerate primers (Additional file 1) based on conserved region were used to amplify the conserved region of *CeWRI*-like gene using cDNAs from leaf, root, and tuber as templates. SMART RACE Amplification kit (Clontech Laboratories, Mountain View, CA) was used to clone 5′ and 3′ fragments of the *CeWRI*-like gene. Full length cDNA sequence was cloned by using primers based on RACE results (Additional file 1).

### Sequence and phylogenetic analyses

In order to find ORFs and to deduce amino acid sequence, open reading frame (ORF) Finder (http://www.ncbi.nlm.nih.gov/gorf/gorf.html) tool was used. NCBI CD-Search tool (http://www.ncbi.nlm.nih.gov/Structure/cdd/wrpsb.cgi) was applied for finding conserved domains in the deduced CeWRI-like protein. Phylogenetic tree of the two-AP2-domain-containing genes was generated by neighbor joining method (with 1000 bootstrap replicates) using MEGA 7.0 software [64].

### Construction of *CeWRI4* over-expression vector and genetic transformation of *Arabidopsis thaliana*

A PCR product of 1423 bp containing complete ORF of *CeWRI4* was digested with *PstI* and *SalI* and then placed between CaMV 35S (35S) promoter and nos terminator of pCambia 2300-35S-nos [65] to construct vector pCambia 2300-35S-*CeWRI4*-nos. Sequencing was performed to ensure that the construct was correct.

WT Arabidopsis seeds were surface sterilized and grown on 1/2 Murashige and Skoog (MS) agar plates containing 3% sucrose in a growth chamber set to 16 h light / 8 h dark (70–80 lm/m^2^/s) at 22 °C after 2 days of stratification at 4 °C. Fifteen-day-old plants were transferred onto soil and grown in a growth chamber at 16 h light/8 h dark (70–80 lm/m^2^/s) at 22 °C. The pCambia 2300-35S-*CeWRI4*-nos vector was introduced into *Agrobacterium tumefaciens* EHA105 which was then used to transform Arabidopsis by floral-dip method [66]. Transgenic plants (T1, T2, T3) were PCR-confirmed by using *NPT II* and *CeWRI4* gene specific primers (Additional file 1).

### Drought tolerance assessment

For PEG-simulated drought tolerance assessment, WT and transgenic Arabidopsis seeds were sown on 1/2 MS agar medium with/without 5% PEG-6000. Germination rate of the WT and transgenic Arabidopsis were scored 6 days after seed sowing. The length of the primary roots was recorded on 2, 4, 6, 8 days after seed germination. Three biological repeat groups were set for each line, each group containing 10 repeat seedlings. For imposing real dehydration stress, two-week-old seedlings, previously germinated on 1/2 MS medium, were transplanted into pots (12 × 12 cm) filled with a mixture of nutrient soil and vermiculite (1:1 ratio). The pots were kept at 22 °C at a 16-h light/ 8-h dark photoperiod. Three biological repeat pots were set for each line, with each pot containing 10 repeat seedlings. Prior to the onset of dehydration stress, the plants were regularly watered and photographed. 20-day-old Arabidopsis plants kept under normal growth condition were given dehydration stress by withholding water. Wilting frequency was determined 15 days after the onset of dehydration stress. Watering was resumed after 22 days of dehydration. Next day, the recovery frequency was scored as the number of surviving plants out of the total plants.

### Water loss measurement

Rosette leaves of 5-week-old seedlings were detached and placed in petri dishes and air-dried to measure transpirational water loss. Weights of detached leaves were measured at different time points during dark exposure. Percentage of water loss was calculated as that follows: (W1-W2)/W1 (W1: Initial leaf weight before water loss; W2: Leaf weight after water loss at different time points).

### Chlorophyll leaching assay

Rosette leaves of 5-week-old Arabidopsis seedlings were immersed in 5 ml of 80% ethanol at room temperature (gently agitating in the dark) for chlorophyll leaching assays. 1 ml of aliquot was taken out at 15, 45, 75, and 105 min for chlorophyll quantification using a UNICO 7202B spectrophotometer (USA) at wavelengths of 663 and 645 nm.

### Physiological analyses of transgenic and WT Arabidopis plants

For measuring photosynthesis-related parameters, 5-week-old transgenic and WT plants were treated with 5% PEG-6000 for 1 day, then Li-6400 photosynthetic system instrument (LI-COR, Inc. Lincoln, USA) was used to measure the rate of stomatal conductance (g_s_), transpiration (T_r_), photosynthesis (P_n_) for leaves of different plants at the same position as described by Wei et al. [67]. Water use efficiency of single leaf (WUE) was calculated as that follows: WUE = photosynthetic rate / transpiration rate.

20-day-old Arabidopsis plants kept under normal growth condition were given dehydration stress by withholding water for 15 days. At the end of the treatment, leaves of transgenic lines and WT were harvested for measurement of MDA, free proline and soluble sugars. For determination of leaf MDA content, thiobarbituric acid method was used as described by Dhindsa and Matowe [68]. Leaf free proline and soluble sugars content was assayed using methods described by Troll and Lindsley [69] and Flood and Priestley [70], respectively.

### Wax extraction and GC-MS analysis

Oil and cuticular waxes were extracted from both leaves and stems of 5 to 6-week-old WT and transgenic Arabidopsis as described by Park et al. [10]. The quantifications were carried out by a GC–MS-QP2010 (Plus) gas chromatograph equipped with flame ionization detector (GC-2010, Shimazu, Tokyo, Japan). Retention times and temperatures for GC were programmed as described by Lee and Suh [18]. Wax composition was determined by comparing peak retention times with those of reference standards. Wax loads were estimated by quantifying the areas of major peaks in comparison with the internal standard. Wax load per unit leaf area and stem area was calculated based on the area of leaves and stems used for wax extraction.

### SEM analysis of deposition of epicuticular wax crystals on Arabidopsis leaves

5-week-old WT and transgenic Arabidopsis plants were drought-stressed for 10 days by withholding water. Mature rosette leaves were sliced into 0.5 cm sections and then fixed in 5% glutaraldehyde and coated with gold particles by using an EICO IB.5 ION coater (Tokyo, Japan). Epicuticular wax crystals on adaxial side of leaves were observed by using JEOLJSM-3690LV scanning electron microscope (Tokyo, Japan).

### RNA extraction and real-time quantitative reverse transcription qRT-PCR

Drought stress was applied to 24-day-old WT and transgenic Arabidopsis plants by treatment with 5% PEG-6000 for 10 h. Control plants were watered without addition of PEG. Rosette leaves from control and treated plants were collected, snap frozen in liquid nitrogen, and stored at − 80 °C. Total RNA was extracted by using the Biospin Plant Total RNA Extraction Kit (Bioflux). To remove any contaminating DNA, the samples were treated with RNase-free DNase (Bioflux). cDNA was synthesized by using the PrimeScript RT reagent Kit (TAKARA). Gene expression was determined by qRT-PCR on Stratagene Mx3005P quantitative PCR system (Agilent Technologies) using BioEasy Master Mix SYBR Green (BIOER). The sequence of the primers used in qRT-PCRs is given in Additional file 1. Specific primer pairs with 100% efficiency and single peaks were selected. *ACTIN2* (At3g18780) was used as reference gene for normalizing the expression. Relative gene expression was calculated by the comparative threshold cycle method.

### Statistical analysis

All experiments were carried out with three replicates. Significant differences were detected by *t* tests using the SPSS software (*P < 0.05; **P < 0.01).

## Supplementary information

**Additional file 1.** Table S1 Primers for PCR and qRT-PCR.

**Additional file 2 **Figure S1 Coding sequence and deduced protein of yellow nutsedge *CeWRI4*. The start and stop codons are marked by black bars.

**Additional file 3.** Figure S2 Phylogenetic tree of the two-AP2-domain-containing genes from different plant species.

**Additional file 4 **Figure S3 Relative expression of *CeWRI4* in different nutsedge tissues. Fresh and tender leaves, roots and tubers were collected from plants growing in the field for RNA extraction and q-PCR.

**Additional file 5 **Figure S4 Relative expression of internal *AtWRI4*. 24-day-old WT and transgenic Arabidopsis plants were treated with 5% PEG-6000 for 10 h. Rosette leaves were harvested for RNA extraction and q-PCR.

**Additional file 6 **Figure S5 qRT-PCR analysis of *DGAT1* involved in oil accumulation in WT and transgenic lines A5, A7, B1, K2: 24-day-old WT and transgenic Arabidopsis plants were treated with 5% PEG-6000 for 10 h. Rosette leaves were harvested for RNA extraction and q-PCR. Error bars indicate ±SD, **P < 0.01, *P < 0.05.

**Additional file 7.** Figure S6 Oil content in leaves of WT and transgenic Arabidopsis grown under normal condition. Ten leaves per replicate were used for measuring weights. Error bars indicate ±SD.

## Data Availability

The datasets supporting results of this study are included in the manuscript and additional supporting files. The sequence for *C. esculentus WRI4* is deposited and available in NCBI Genbank as accession MW039149.

## Abbreviations

APETALA2/EREBP: Ethylene Responsive Element Binding Protein
BCCP2: Biotin carboxyl carrier protein 2
BSTFA: Bis-N, N trimethylsilyl trifluoroacetamide
CER1: ECERIFERUM 1
CER4: ECERIFERUM 4
Chl: Chlorophyll
CoAs: Coenzyme As
DAG: Days after germination
DGAT1: Diacylglycerol acyltransferase 1
FW: Fresh weight
GC-MS: Gas Chromotography-Mass spectrometry
KCS1: 3-ketoacyl-CoA synthase 1
LACS1: Long-chain acyl-coA synthase 1
MDA: Malondialdehyde
MS: Murashige and Skoog
PDHE1α: Pyruvate dehydrogenase E1 alpha
PEG: Polyethylene glycol
PKp-β1: Plastidic pyruvate kinase beta subunit 1
qRT-PCR: Quantitative reverse transcription Polymerase Chain Reaction
q-RT-PCR: Quantitative RT-PCR
RACE: Rapid amplification of cDNA ends
SEM: Scanning electron microscopy
TAG: Triacylglycerol
UTR: Untranslated region
VLCFAs: Very-long-chain fatty acids
WSD1: Wax ester synthase/diacylglycerol acyltransferase 1
WT: Wild type
WUE: Water use efficiency
