# Supplementary material for: Yellow nutsedge WRI4-like gene improves drought tolerance in Arabidopsis thaliana by promoting cuticular wax biosynthesis
Source: BMC Plant Biol. 2020 Oct 31;20:498. doi: 10.1186/s12870-020-02707-7 (PMC7603781; doi:10.1186/s12870-020-02707-7)
Supplement: Supplementary file 1 — Additional file 1. Table S1 Primers for PCR and qRT-PCR. [file 12870_2020_2707_MOESM1_ESM.docx]

| **Primer name** | **Sequence information** |
| --- | --- |
| BCCP2 FP | 5‘-AAGGAGCGGTTTGGTGAACTT |
| BCCP2 RP | 5‘-CGCAACCTCCAGAATGCAGA |
| PIPK-β1 FP | 5‘-CATCGGTAGGGATGTGGTCG |
| PIPK-β1 RP | 5‘-TGGTGTGCTGACTCCTCTCT |
| PDHE1αFP | 5‘-GAGGCCAAGGTGGATCCAT |
| PDHE1αRP | 5‘-CAAAGCCACCAAGCATGTTG |
| DGAT1 FP | 5‘-TGGATTCTGCTGGCGTTACTAC |
| DGAT1 RP | 5‘-AGCCTATCAAGATCGACGAACTCT |
| LACS1 FP | 5‘-CTGAACTCACTGAAGAAGTC |
| LACS1 RP | 5‘-CACACACATCATCGCAACAC |
| WSD1 FP | 5‘-GCTTGGTGGTTGTTTGTTGG |
| WSD1 RP | 5‘-GACAATCTTGTCTACGTAGC |
| KCS1 FP | 5‘-GTAAGCACGGAAAACATAACCCTA |
| KCS1 RP | 5‘-CGTCTGATCCTTTATGTGTTCGA |
| CER1 FP | 5‘-AGGTCGACAGGGAGACCAAC |
| CER1 RP | 5‘-ATAAGCGCTGCCATCAACAC |
| CER4 FP | 5‘-ACCGTGGACCAACAAAGAAG |
| CER4 RP | 5‘-GCAATCAAGTAGCGTATGGTCA |
| ACTIN2 FP | 5‘-TGTGACAATGGTACCGGTATGG |
| ACTIN2 RP | 5‘-GCCCTGGGAGCATCATCTC |
| degenerate FP | 5‘-GGTTCGAGGCCCACYTNTGGGAYAA |
| degenerate RP | 5‘-TCGATGGCGGCCADRTCRTANGC |
| 5’-RACE | 5‘-CGGCGGCTTCTTCTTCTGTTGCGTATG |
| 3’-RACE | 5‘-TTGGGATAAGAACAGTTGGAATGAGAC |
| *CeWRI4* full length FP | 5‘-TCCCCTCTCGTTTTAGCTCTCCTTCATTTC |
| *CeWRI4* full length RP | 5‘-GGCAAGCAGTGGTATCAACGCAGAGTAC |
| vector construction FP | 5‘-ATAGGATCCTCCCCTCTCGTTTTAGCTCTCCTTCATTTC |
| vector construction RP | 5‘-ATACTGCAGGGGCAAGCAGTGGTATCAACGCAGAGTA |
| *CeWRI4* qRT-PCR FP | 5‘-GTTCGAGGCGCATTTGTGGGATAAG |
| *CeWRI4* qRT-PCR RP | 5‘-TCCGTCGCATATGTTCCAAGGTAGAGA |
| NPTII FP | 5‘-CCGGCCGCTTGGGTGGAGAGG |
| NPTII RP | 5‘-CGCCCAATAGCAGCCAGTCCCTTC |

拟南芥WRI4引物序列
