# Supplementary figures and images for: Yellow nutsedge WRI4-like gene improves drought tolerance in Arabidopsis thaliana by promoting cuticular wax biosynthesis
Source: BMC Plant Biol. 2020 Oct 31;20:498. doi: 10.1186/s12870-020-02707-7 (PMC7603781; doi:10.1186/s12870-020-02707-7)

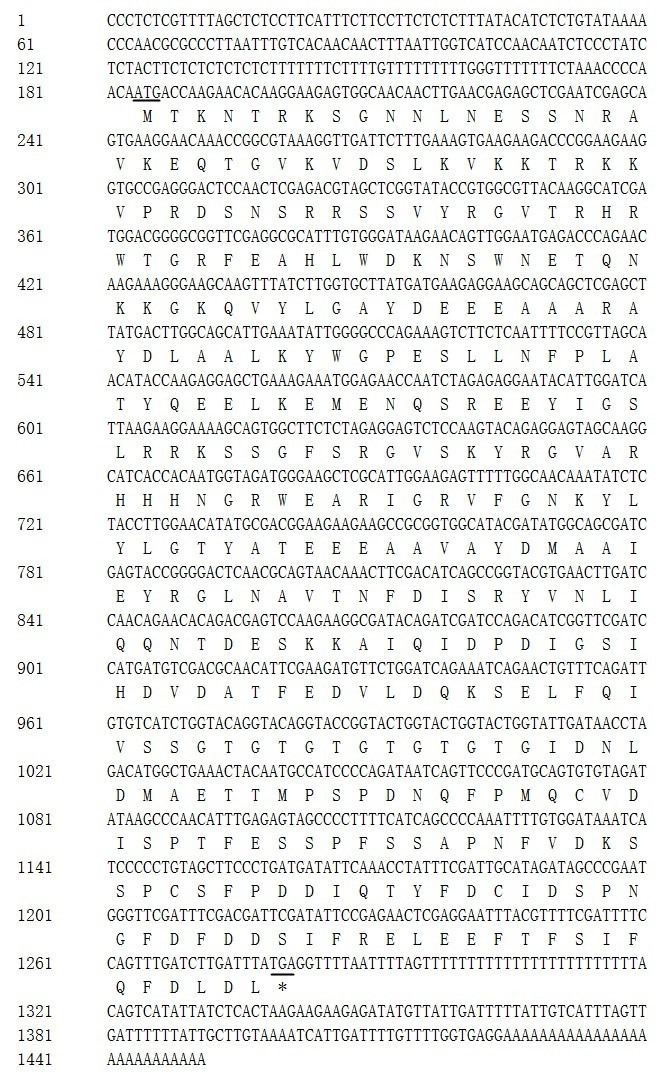

Supplement: Supplementary file 2 — Additional file 2 Figure S1 Coding sequence and deduced protein of yellow nutsedge CeWRI4. The start and stop codons are marked by black bars. [file 12870_2020_2707_MOESM2_ESM.docx]

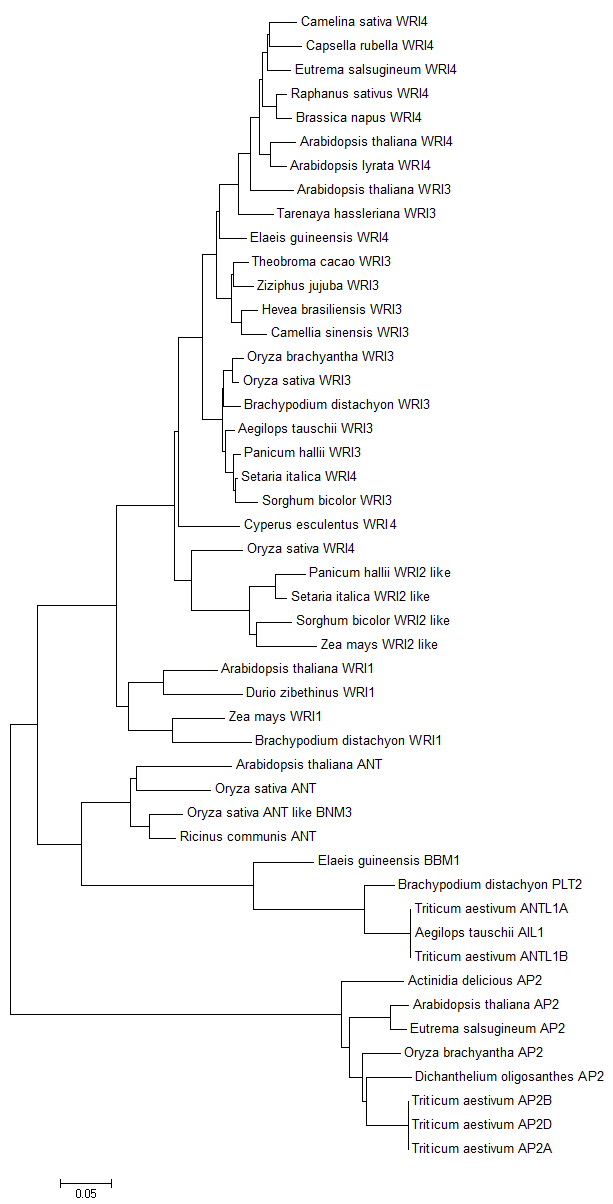

Supplement: Supplementary file 3 — Additional file 3. Figure S2 Phylogenetic tree of the two-AP2-domain-containing genes from different plant species. [file 12870_2020_2707_MOESM3_ESM.docx]
