# Supplementary material for: Yellow nutsedge WRI4-like gene improves drought tolerance in Arabidopsis thaliana by promoting cuticular wax biosynthesis
Source: BMC Plant Biol. 2020 Oct 31;20:498. doi: 10.1186/s12870-020-02707-7 (PMC7603781; doi:10.1186/s12870-020-02707-7)
Supplement: Supplementary file 4 [file 12870_2020_2707_MOESM6_ESM.docx]

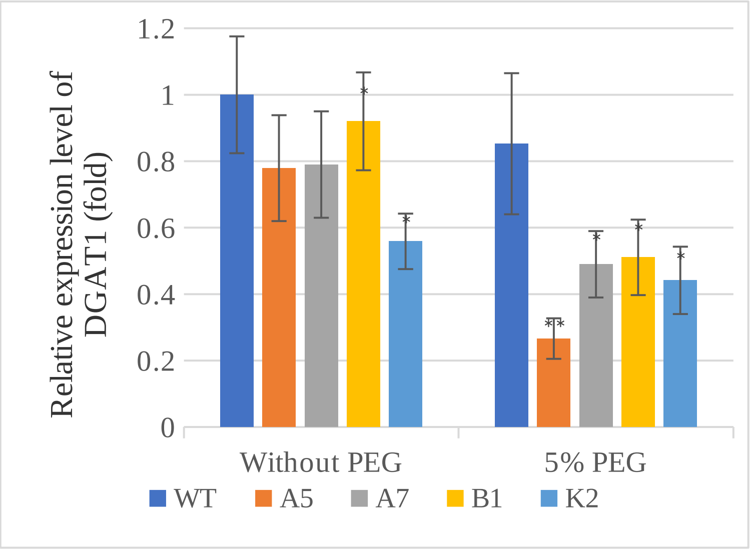

Relative expression level of DGAT1 (fold)
1.2
1
0.8
0.6
0.4
0.2
0
*
*
**
*
*
*
Without PEG
5% PEG
WT
A5
A7
B1
K2
