# Supplementary material for: Yellow nutsedge WRI4-like gene improves drought tolerance in Arabidopsis thaliana by promoting cuticular wax biosynthesis
Source: BMC Plant Biol. 2020 Oct 31;20:498. doi: 10.1186/s12870-020-02707-7 (PMC7603781; doi:10.1186/s12870-020-02707-7)
Supplement: Supplementary file 5 [file 12870_2020_2707_MOESM7_ESM.docx]

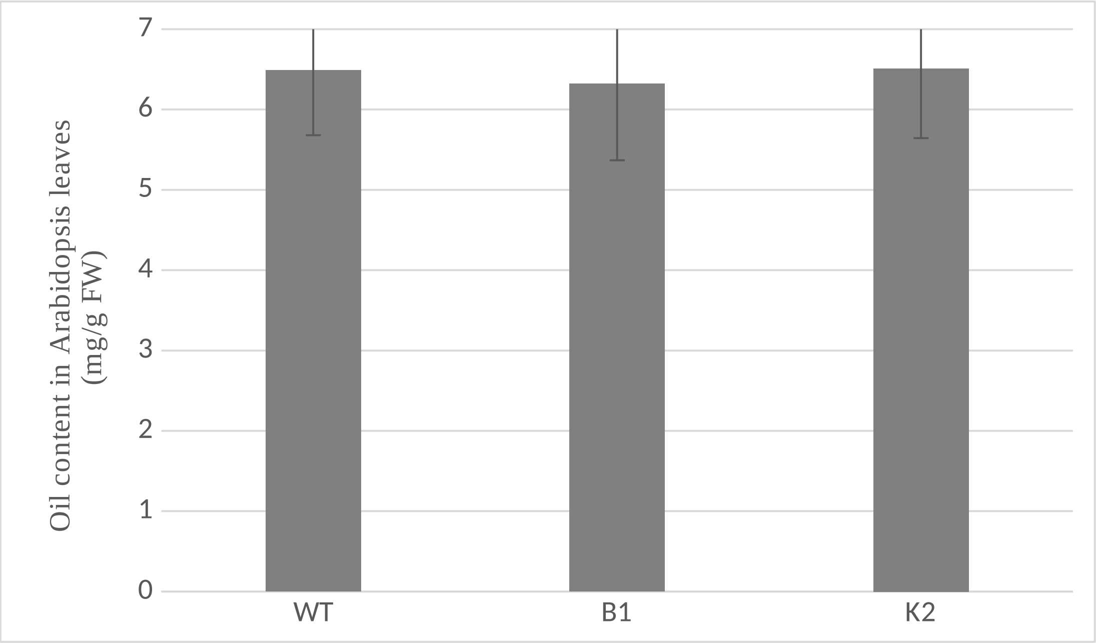

Oil content in Arabidopsis leaves (mg/g FW)
7
6
5
4
3
2
1
0
WT
B1
K2
